
JOURNAL INFORMATION
==============================
NLM Title Abbreviation: ScientificWorldJournal
Journal ID: ScientificWorldJournal
NLM Journal ID: 101131163
Journal ID: TSWJ

The Scientific World Journal

ISSN: 2356-6140
EISSN: 1537-744X
Publisher: Wiley

ARTICLE INFORMATION
==============================
PMCID: PMC5917156
PMID: 17072490
DOI: 10.1100/tsw.2006.245
Article ID: 464956
Article version: 1
Subjects: Clinical Images

Early Satiety — An Unusual Cause

Weir Robin AP 1 *
McMillan Nigel 2
1 Department of Cardiology Western Infirmary, Glasgow, UK
2 Department of Radiology Western Infirmary, Glasgow, UK
*Corresponding author.Academic Editor: Daniel Batlle

Publication date: 2006
Electronic publication date: 2006 Oct 25
Volume: 6
First page: 1373
Last page: 1374
Received 2006 Sep 15; Accepted 2006 Oct 15
Copyright: Copyright © 2006 Robin AP Weir and Nigel McMillan.
Copyright year: 2006
License: This is an open access article distributed under the Creative Commons Attribution License, which permits unrestricted use, distribution, and reproduction in any medium, provided the original work is properly cited.
License URL: https://creativecommons.org/licenses/by/3.0/

Keywords: pancreatic pseudocyst, cardiac magnetic resonance, imaging techniques

==============================
Clinical Image
*TheScientificWorldJOURNAL* (2006) 6, 1373–1374
ISSN 1537-744X; DOI 10.1100/tsw.2006.245

TheScientificWorldJOURNAL
www.thescientificworld.com

### Early Satiety — An Unusual Cause

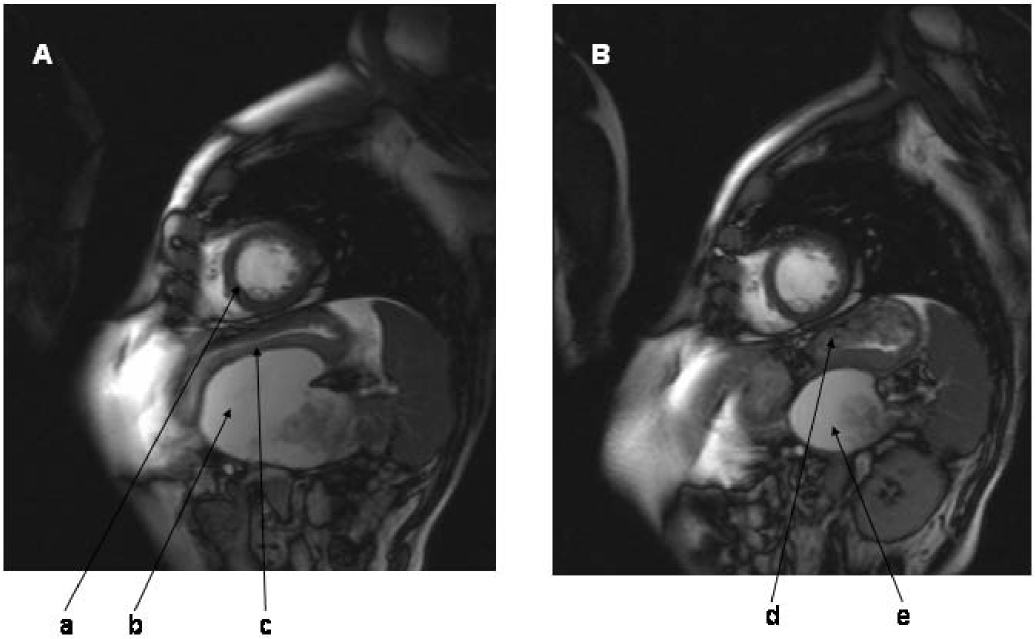

**Figure 1A.** Short-axis retro cine image through the mid-left ventricular cavity (a). A large pseudocyst (b) is seen within the lesser peritoneal sac, compressing the empty stomach (c). **Figure 1B.** The same MRI slice taken after three months of conservative treatment shows a significant reduction in the size of the pseudocyst (e). More space is afforded for gastric expansion as demonstrated by visible food contents within the stomach (d).

Robin Anthony Patrick Weir[1,*] and Nigel McMillan[2]

*[1]Department of Cardiology and [2]Department of Radiology, Western Infirmary, Glasgow, Scotland, U.K.*

**E-mail**: robinweir75@hotmail.com; nigel.mcmillan@northglasgow.scot.nhs.uk

*Received September 15, 2006; Accepted October 15, 2006; Published October 25, 2006*

**KEYWORDS:** pancreatic pseudocyst, cardiac magnetic resonance, imaging techniques

A 50-year-old man with a history of alcohol excess presented 4 weeks after an acute flare of his known chronic pancreatitis with an inferoposterior myocardial infarction (MI). Systemic enquiry revealed recent weight loss and early satiety. Following percutaneous treatment of the acute infarct, the patient underwent a cardiac magnetic resonance scan (CMR) as part of a clinical trial (1.5T Siemens Sonata). Cine images through the mid-left ventricle revealed an extensive pancreatic pseudocyst measuring 9.1 × 6.9 cm,

*Corresponding author.
©2006 with author.
Published by TheScientificWorld, Ltd.; www.thescientificworld.com displacing the (empty) stomach anteriorly and providing a possible explanation for the early satiety (Fig. 1A).

The most appropriate management of pancreatic pseudocyst — open surgery, percutaneous drainage, or conservative management — is subject to ongoing debate[1,2,3]. Conservative management, at least in the short term, was deemed the most pragmatic approach in view of the recent extensive MI. A second CMR scan 3 months later (Fig. 1B), performed 30 min after a meal — gastric contents visible — indicated that the pseudocyst had reduced in size, affording more room for gastric expansion. The patient's weight had improved and the early satiety had resolved.

While gastric compression is a recognised anatomical consequence of a large pseudocyst, it is rarely clinically symptomatic. This case clearly displays the anatomical and functional consequences of gastric compression by a moderate pancreatic pseudocyst, and their resolution with conservative management only.

### REFERENCES

1. Varadarajulu, S. (2006) Non-operative management of pancreatic pseudocysts: there is still a role. *Ann. Surg*. **244(1),** 161–162.
2. Andersson, B., Nilsson, E., Willner, J., and Andersson, R. (2006) Treatment and outcome in pancreatic pseudocysts. *Scand. J. Gastroenterol*. **41(6),** 751–756.
3. Schlosser, W., Siech, M., and Beger, H.G. (2005) Pseudocyst treatment in chronic pancreatitis--surgical treatment of the underlying disease increases the long-term success. *Dig. Surg.* **22(5),** 340–345.

**This article should be cited as follows:**

Weir, R.A.P. and McMillan, N. (2006) Early satiety — an unusual cause. *TheScientificWorldJOURNAL* **6**, 1373–1374. DOI 10.1100/tsw.2006.245.
